# Evaluation of flood preparedness in government healthcare facilities in Eastern Province, Sri Lanka

**DOI:** 10.1080/16549716.2017.1331539

**Published:** 2017-06-14

**Authors:** Jessica M. Farley, Inoka Suraweera, W. L. S. P. Perera, Jeremy Hess, Kristie L. Ebi

**Affiliations:** ^a^ Department of Global Health, University of Washington, Seattle, WA, USA; ^b^ Environmental and Occupational Health Directorate, Ministry of Health, Colombo, Sri Lanka; ^c^ WHO Health Emergencies Programme, World Health Organization, Colombo, Sri Lanka

**Keywords:** Climate change, flood, natural disaster, preparedness, health system, Sri Lanka

## Abstract

**Background**: Sri Lanka is vulnerable to floods and other hydro-meteorological disasters. Climate change is projected to increase the intensity of these events.

**Objective**: This study aimed to assess the flood preparedness in healthcare facilities in Eastern Province.

**Design**: This was a cross-sectional, descriptive, mixed methods study conducted in Trincomalee District. Surveys were conducted in 31 government healthcare facilities, using a pre-tested, structured questionnaire covering the last 5 years. Seven in-depth interviews were conducted with randomly selected Medical Officers in Charge or their equivalent, and 3 interviews were conducted with Medical Offices of Health.

**Results**: Two general hospitals, 3 base hospitals, 11 divisional hospitals, and 15 primary care units were included. Six respondents (19.4%) reported flooding in their facility, and 19 (61.3%) reported flooding in their catchment area. For the health workforce, 77.4% of respondents reported not enough staff to perform normal service delivery during disasters, and 25.5% reported staff absenteeism due to flooding. Several respondents expressed a desire for more disaster-specific and general clinical training opportunities for themselves and their staff. Most respondents (80.7%) reported no delays in supply procurement during weather emergencies, but 61.3% reported insufficient supplies to maintain normal service delivery during disasters. Four facilities (12.9%) had disaster preparedness plans, and 4 (12.9%) had any staff trained on disaster preparedness or management within the last year. One quarter (25.8%) of respondents had received any written guidance on disaster preparedness from the regional, provincial, or national level in the last year.

**Conclusions**: While there is a strong health system operating in Sri Lanka, improvements are needed in localized and appropriate disaster-related training, resources for continuing clinical education, and investments in workforce to strengthen flood and other disaster resilience within the government healthcare system in the study district.

## Background

Climate change is a reality uncontested in the scientific community, with effects projected to have dramatic consequences for the environment and human health, from more extreme weather events to changes in the distribution and incidence of vector-borne infectious diseases and increases in heat-related illnesses. These will put pressure on health systems.

The Intergovernmental Panel on Climate Change (IPCC) cites robust evidence that warming during the twenty-first century will put more people at risk of being affected by floods [1]. The Lancet Commission on climate change posits that ‘the ability of health systems to respond effectively to the direct and indirect health effects of climate change is a challenge worldwide, especially in many low-income and middle-income countries, which suffer from disorganised, inefficient, and under-resourced health systems’ [2, p. 1704]. Building resilience and adaptive capacity to climate-related hazards and disasters is part of the United Nations Sustainable Development Goals [3]. Health facilities, often the first line of contact for disaster victims, are a major component of disaster preparedness and response. It is therefore imperative to understand the functional capacity of these facilities to prepare for and respond to hydro-meteorological hazards.

A flooding event does not necessarily result in a disaster. ‘Floods become disasters when they are of unusual proportion, occur in unusual places, or occur unexpectedly’ [4, p. 5]. There are no disasters without human components, so it is essential to understand the human systems in place in high-risk areas and the resilience and capacity of these systems to prepare and manage risks.

Sri Lanka is at risk for numerous types of extreme events and disasters, including floods, droughts, tsunamis, cyclones, coastal erosion, sea level rise, and landslides. The 2004 tsunami, one of the deadliest disasters in human history, killed over 30,000 people and left over 5000 missing in Sri Lanka [5]. Other, less intensive climate-related disasters, however, comprise the bulk of natural disasters in Sri Lanka. Among these, floods are the most common, and the third most common type of recorded disaster between 1974 and 2007, behind epidemics and animal attacks [6]. For average annual flood exposure in proportion to population, Sri Lanka ranks eleventh in the world [7]. Although mortality is low, floods annually displace hundreds of thousands of people across the country and have widespread effects; in May 2016, heavy rainfall and subsequent flooding and landslides displaced nearly 225,000 people [8].

Mortality from weather-related disasters increased in Sri Lanka from 1974 to 2007 by 1.70%, while damage to houses increased by 5.68% [9]. Projections suggest that one-day heavy rainfall events will increase over the coming decades, which can lead to rapid flooding. A spatio-temporal analysis of rainfall in the Eastern Province of Sri Lanka from 1980 to 2010 showed that while the number of rainy days decreased, rainfall itself increased, suggesting increased intensity of rainfall events [10]. The authors concluded changes in flooding and droughts could probably be attributed to temporal changes in rainfall distribution [10].

The proportion of households living below the poverty line is correlated with damage to houses due to flooding [9]. Trincomalee District, the site of this study, has a mean monthly household income of US$ 240 (Rs 34,577) [11]. Nine percent of the population is below the poverty line in Trincomalee District, suggesting increased risk and decreased resilience for disasters.

At the national level, health services in Sri Lanka function under the Ministry of Health, Nutrition, and Indigenous Medicine. This central Ministry of Health is responsible primarily for the protection and promotion of population health. Its key functions include setting policy guidelines, management of teaching and specialized institutions including the country’s network of teaching hospitals, medical and paramedical education, bulk purchasing of medical requisites, and providing technical support to the Provincial Ministries.

Sri Lanka has 25 districts organized into nine provinces. Since the implementation of the Provincial Councils Act in 1989, government administration is decentralized to the provinces; provincial councils in turn oversee districts, which are administered by District Secretariats. The coordinating bodies for health are the Provincial Directors of Health Services (PDHS) at the provincial level and the Regional Directors of Health Services (RDHS) at the district level.

Coordination for disaster and climate change response in Sri Lanka is stratified across different government ministries at different levels. Overall disaster preparedness and response falls under the purview of the Ministry of Disaster Management, with relevant coordinating units in other ministries. In the Ministry of Health, the coordinating body is the Disaster Preparedness and Response Division (DPRD), which acts as a focal point on the healthcare aspects of all emergencies and is responsible for disseminating guidance to the PDHS. The DPRD coordinates on disaster response with the Disaster Management Center (DMC), the lead agency for disaster management. The focal point for climate change in Sri Lanka is the Climate Change Secretariat, housed in the Ministry of Mahaweli Development and Environment, and the Directorate of Environmental and Occupational Health functions as the focal point for climate change in the health sector.

Curative public facilities include the National Hospital in Colombo, teaching and specialized hospitals, provincial and district general hospitals, base hospitals, divisional hospitals, and primary medical care units (PMCUs), which are the smallest type of facility responsible for providing first-line care. In addition, there are Medical Offices of Health responsible for implementing preventive health programs in their divisions. They also serve as an organizing mechanism, in collaboration with the divisional secretary, for flood response, while RDHS offices coordinate with the District Secretariats. Their duties in this area include setting up camps and providing aid for displaced people, monitoring water and sanitation, and mosquito control during and post-flood.

## Methods

This was a cross-sectional, descriptive, mixed methods study using a structured questionnaire and in-depth interviews. The study setting was public healthcare facilities (curative) and Medical Offices of Health (preventive) in Trincomalee District, Eastern Province, Sri Lanka. The objective of this study was to assess the functional preparedness of public healthcare facilities at all levels in Trincomalee District for flood events, as well as their general preparedness for disasters.

Trincomalee District covers 2727 square kilometers, with a population in 2012 of 378,182. The district has 34 curative health facilities and 11 Medical Offices of Health (preventive health) areas. In 2016, the Trincomalee District curative health facilities included 3 base hospitals, 11 divisional hospitals, 18 PMCUs overseen by the PDHS, and 2 general hospitals overseen directly by the Ministry of Health.

Participants surveyed were Medical Officers in Charge (MOIC) or equivalents. A pre-tested, structured questionnaire adapted from the World Health Organization (WHO) Hospital Safety Index [12] was administered face-to-face for 31 of the institutions (Appendix). Among the three not included, medical officers were on leave at two, and the third facility was not functioning. The questionnaire investigated flood history within the health facility and in the surrounding area, communication on disaster management with the Provincial and Regional Director of Health Services offices, staff capacity, procurement procedures, and disaster preparedness. Among these 31 health facilities, 7 were selected using a stratified random sample for additional in-depth interviews with the same initial respondent. An additional 3 interviews were conducted with representatives from Medical Offices of Health, for a total of 10 interviews. Informed consent was obtained from all interview participants, and interviews were tape recorded and transcribed for analysis.

This study received a determination of non-research from the University of Washington Human Subjects Division of the Institutional Review Board (IRB). It was approved after full committee review by the Ethical Review Committee at Trincomalee General Hospital in Eastern Province, Sri Lanka.

Stata13 was used to analyze quantitative data. Qualitative interview data were analyzed in ATLAS.ti.

## Results

Table 1 shows characteristics of the studied health facilities. Within the last five years, 6 facilities (19.4%) directly experienced flooding (defined as standing water lasting more than one day in buildings or grounds), while 19 facilities (61.3%) experienced flooding in their catchment area. Among the 6 facilities that experienced flooding directly, the mean number of floods was 4.7 (SD = 3.2, min = 1, max = 10), with an average length of 25.2 days (SD = 33.2, min = 3, max = 90), and cleanup lasted an average of 4.3 days after the end of the event (SD = 5.5, min = 1, max = 15). All six facilities reported impaired road access to and from their facility at least once, while four reported power outages, and two reported phone lines being down.

**Table 1.** Characteristics of facilities surveyed (n = 31).

Variable	n (%), mean (SD), or median (IQR)	
*Health facilitycharacteristic*		
Type of facility		
Line Ministry hospital^a^	2	(6.5%)
Base hospital	3	(9.7%)
Divisional hospital	11	(35.5%)
Primary Medical Care Unit	15	(48.4%)
Mean bed capacity		
Line Ministry hospital	317.5	(SD = 116.7)
Base hospital	124.7	(SD = 90.7)
Divisional hospital	37.4	(SD = 18.7)
Primary Medical Care Unit^b^	0.3	(SD = 0.9)
Mean patients per day (in- and out-patient)		
Line Ministry hospital	575.0	
Base hospital	550.0	
Divisional hospital	150.1	
Primary Medical Care Unit	105.2	
Facility damaged in 2004 tsunami	5	(16.1%)
Generator	18	(58.1%)
Running water in facility	29	(93.6%)
*Respondent characteristics*		
Current position		
Registered Medical Officer or MOIC	26	(83.9%)
Chief Administrative Officer	1	(3.2%)
Other	4	(12.9%)
Mean years respondent worked in facility	3	(IQR = 2–5)
Minimum	0.08	
Maximum	17	
Years respondent had practiced as physician	13	(IQR = 3–23)
Have an existing disaster protocol or plan	4	(12.9%)
Disaster training for any facility staff within last 1 yr	4	(12.9%)
*Flooding history*		
Floods in facility (structure or premises)	6	(19.4%)
Floods in catchment area	19	(61.3%)

Notes: ^a^Hospital controlled directly by the Ministry of Health.

^b^With infrequent exceptions, PMCUs do not have inpatient capacity.

### Leadership/governance

Communication (written or verbal) regarding disaster preparedness was rare with both the PDHS and RDHS offices. Because the RDHS serves as the intermediary between health facilities and the PDHS, only two facilities (6.5%) reported any disaster communication directly with the PDHS in the last year. Twelve (38.7%) facilities reported communication on disaster preparedness with the RDHS in the last year; one facility reported communication ≥ 12 times, nine reported 1–5 times, and two reported 6–11 times. Ten of the 12 facilities (83.3%) reported that they were somewhat or completely satisfied with both the frequency and quality of communication. Respondents were also asked if they had received any instructive documents on disaster preparedness of any sort from the PDHS, RDHS, or DMC (Disaster Management Centre) within the last year. Three facilities (9.7%) reported receiving documents from the PDHS, three (9.7%) from the RDHS office, and two (6.5%) from the DMC. Both facilities receiving documents from the DMC were larger hospitals.

### Emergency preparedness

Only four facilities (12.9%) had an emergency preparedness plan, all of which were in writing. One facility performed an exercise or drill of the plan in the last year. Four respondents (12.9%) reported anyone in their facility having attended disaster training of any sort in the last year. Most respondents had never received training, or had received only one during their medical education. Topics of interest for future training included emergency coordination and management, injury treatment, and rescue and evacuation procedures. Among those who had received non-academic training, many attended International Committee of the Red Cross (ICRC) trainings conducted during the civil conflict. In interviews, PMCU and base/divisional hospital respondent assessment of emergency preparedness was mixed. Responses suggested a moderate level of infrastructure and a high personal willingness for disaster response, but also a shortage of knowledge specific to weather-related disasters and disaster coordination. This was captured by one respondent:

We can handle it, because everybody is going to do their best, right, people will come to work, staff will come to work, and they will do as much as they can, but if you take like, are you trained? Do you have enough stuff? Then no. But we will do everything we can. Staff will do everything here; we will use every drug we have, everything we know we will do, but we are not trained to do it.

During interviews, older doctors expressed greater confidence in handling disaster situations due to their work experience and practice during the conflict. One respondent completed most of his internship (equivalent: residency) in a conflict area:

so that experience [in training hospital] and during the conflict, the ethnic conflict, that time also I think around 3 months, we worked in the surgical team managing these casualties brought by the ships, so around 4000 people, wounded people, we managed.

Continuous training of any sort was infrequent. Up-to-date medical information is not disseminated in a way that many respondents found accessible, and their university medical education was the last that several had received. The rural nature of many facilities in this area amplified this, coupled with a lower level of post-war infrastructure in Trincomalee District as compared to other parts of the country. Self-driven knowledge acquisition was a theme cited frequently in interviews:

For the doctors in the periphery they are like just dumped… there are sort of no updates; if we are interested in [more information] we get it ourselves by the Internet, we don’t get the proper protocol, even for the clinical management we don’t get the protocols.

### Workforce

Facilities reported their numbers of clinical staff (defined as doctors, nurses, and midwives) and minor staff (attendants, drivers, dispensers, and cleaners). PMCUs typically had one physician, and an average of 5.1 total staff members. Divisional hospitals reported 5.5 clinical and 17.5 total staff on average. Base hospitals reported 62.3 clinical staff and 115.0 total staff, with considerable variation (n = 3, min/max 10–90 and 27–167, respectively). Line ministry hospitals also showed variation, with an average of 438 clinical staff and 727 total staff (n = 2, min/max 192–684 and 437–1017, respectively).

When asked if they had enough staff to perform normal service delivery during a flood or other weather emergency, 24 facilities (77.4%) reported that they did not; these were 12 of the 15 PMCUs, 10 of the 11 divisional hospitals, and 2 of the 3 base hospitals. Both line ministry hospitals reported sufficient staff. In interviews, several respondents in PMCUs and base or divisional hospitals remarked that they did not have enough staff for even non-emergency periods. Eight facilities (25.8%) reported staff absenteeism due to flooding in the past five years, of which six said this had created a problem in service delivery. However, only three facilities (9.7%) reported that there were ever times when services were not available due to storms or floods, with the most commonly cited reason being impaired or blocked road access to the health facility. Many respondents described their staff doing ‘whatever it takes’ to get to work during floods and working long hours to see all patients. As one physician in a facility that regularly experiences flooding explained, ‘they would somehow come, because they know that if one person doesn’t come, there is no person to cover for them.’

Human resource shortages were present at many facilities. Most commonly, there were shortages of nursing officers, leaving physicians as the only or primary trained clinical staff in the facility. Because of this shortage, non-clinical staff often perform minor procedures (e.g. wound care, IV insertion) and dispense medications in place of a dispenser. The majority of PMCUs had just 1 doctor, seeing an average of just over 100 patients per day. Many respondents described the all-encompassing nature of their work; they often live on hospital premises, and are left with little time to read circulars, seek additional training, or address long-term issues of resiliency and preparedness. Two respondents explained their experiences:

Only 2 doctors, 15 nights [on-call], you have to always be inside of the hospital, you have to attend the patient any time and it is really stressful to the patient, because when you are staying in the quarters it means you are inside of a hospital, that’s a mentality that myself I know.

They usually send some circular or something, I don’t know when it was sent, because here there are only doctors working with this station, no? So most of them won’t have time to participate in that program, that’s the problem. Most of the people here [minor staff] are uneducated; when we go for that program the patients create unnecessary problems, so most of the time we [doctors] like to be in the hospital. You can see all the roads are broken; the patients came here with difficulty, so we also like to be here in the hospital.

As minor staff made up the majority of the workforce at most smaller facilities, the strengths and weaknesses of these staff also emerged as a theme. Some respondents mentioned that they benefited greatly from the experience of minor staff; for example, some are skilled at small procedures, and most are from the local area and therefore have a better understanding of the community. Others thought better training of minor staff would reduce overload on clinical staff, and would better position facilities to perform everyday service delivery and be better prepared for disaster situations. As one respondent explained, many minor staff ‘can’t even dress a wound. So they should have something like that, like generally they should teach everybody, if you are working in a hospital, how to manage small things.’

### Supplies and facility infrastructure

Twenty-nine facilities (93.6%) had first-aid kits. The majority (80.7%) reported no delay in supply procurement during weather emergencies, while two (both divisional hospitals) reported delays of less than a day and four (one base hospital, three divisional) reported delays of one to six days. Several respondents reported sharing supplies with other nearby facilities during emergency and non-emergency periods. Nineteen respondents (61.3%) reported they did not believe their facility had enough medical equipment and supplies to maintain normal service delivery during a weather emergency. Supplies listed as lacking included medical equipment (electrocardiogram monitors, oxygen cylinders, and other instruments) as well as general facility equipment such as fridges, beds, trolleys, and perimeter fencing or walls.

### Overall flood preparedness

**Figure 1.**
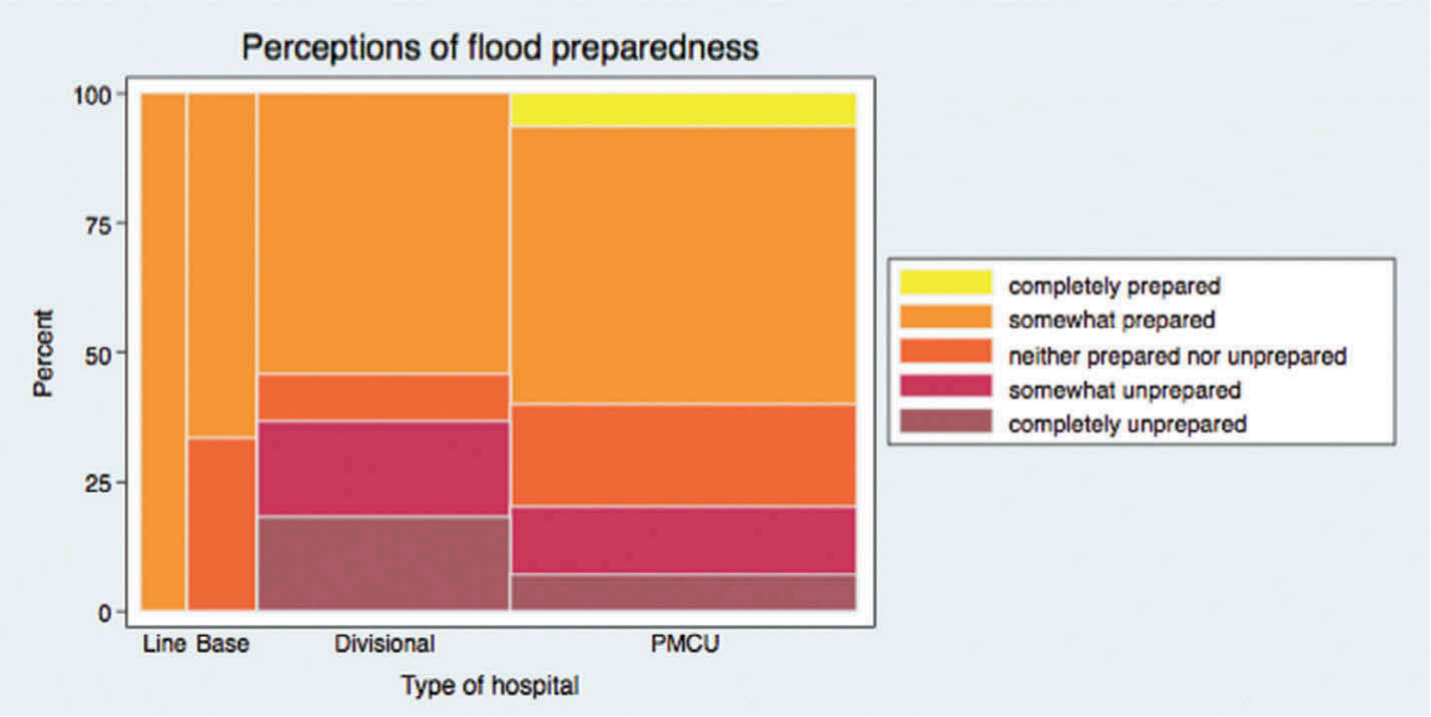
Perceptions of overall preparedness for a major flood.

Respondents were asked how prepared they felt their health facility was overall for a major flood event using a five-point scale: Completely unprepared (1), Somewhat unprepared (2), Neither prepared nor unprepared (3), Somewhat prepared (4), and Completely prepared (5). Figure 1, with the column widths proportional to number of facilities, shows that over half of each type of facility ranked themselves as at least somewhat prepared. However, responses of ‘somewhat’ or ‘completely’ unprepared were reported among some divisional hospitals and PMCUs.

In interviews, preparedness was perceived as important both within the health facility and for the community at large. As one MOIC described: ‘so we have to educate… we have to assess the people and educate them about the disaster. So, to reduce their panic, that is important.’

Three in-depth interviews were conducted with Medical Officers of Health. Their responses suggested their ability to respond to floods was mostly satisfactory, with enough resources (sometimes brought in from outside the district) to manage. Improvements suggested echoed the responses from curative facilities: dealing with staff shortages and lack of qualified dispensers during emergencies, and a desire for more training to strengthen preparedness. Community trainings were also suggested. A respondent from a Medical Office of Health in a flood-prone area characterized the public’s response in their catchment area: ‘there is a lack of social mobilization. People are not aware how to act during a disaster; they are concerned about food and aid, but not about sanitation or mosquito-borne illnesses.’

### Climate change awareness

Respondents were asked questions on their perceptions of climate change (Table 2). Two reported that they had received information from any governing body on climate change and health issues. Awareness about climate change was mixed among respondents. While just under two-thirds responded that climate change was occurring in Sri Lanka, over 90% of respondents acknowledged that changes in climate (seasonal or systematic) can affect human health. Respiratory tract infections, viral fever, diarrhea, conjunctivitis, and asthma were cited as frequent problems associated with the rainy season, and rashes and other skin problems, chicken pox, and allergies with the hot season. Some respondents only saw it as a problem in other parts of the country, such as the west coast, or in the mountains where landslides occur after heavy rains.

**Table 2.** Climate change perceptions.

Climate change questions	n (%)	
Do you think flood and storm events happen more frequently than they used to?		
Yes	10	(32.3%)
No	18	(58.1%)
Don’t know	3	(9.9%)
Do you think flood and storm events are stronger or more intense than they used to be?		
Yes	9	(29.0%)
No	16	(51.6%)
Don’t know	6	(19.5%)
Do you think that climate change is occurring in Sri Lanka?		
Yes	20	(64.5%)
No	7	(22.6%)
Don’t know	4	(12.9%)
Do you think changes in climate can affect human health?		
Yes	28	(90.3%)
No	1	(3.2%)
Don’t know	2	(6.5%)

## Discussion

The WHO outlines 10 components for a climate-resilient health system, rooted in the 6 WHO health systems building blocks: financing, service delivery, essential medical products and technologies, health information systems, health workforce, and leadership and governance [13]. This study focused primarily on service delivery and health workforce. The framework calls for an adequate baseline level of qualified staff to create a climate-resilient system. Many respondents felt they were understaffed and that staff in their facilities were overworked, leaving time only for direct clinical care. Getting qualified nurses to Trincomalee District appears to be a major challenge, possibly due to the rural location of many peripheral hospitals and the lack of a nursing college in the district.

Lack of continuing professional education was a common thread throughout this study. Respondents cited the Internet or private courses as their best options for obtaining additional training or certifications. The urban/rural dynamic is also an important consideration. Trincomalee District is far from the capital, and many facilities are geographically isolated. Physicians in rural areas are frequently at a disadvantage when it comes to information access due to professional and geographic isolation, less Internet and other information and communication technology infrastructure, and limited professional development opportunities [14–16].

The lack of training on disaster preparedness and management is one facet of this information shortage. A minority of respondents, in either preventive or curative settings, had ever received training on preparedness or response for any sort of disaster situation, and plans and protocols were unclear. Similar situations were found in hospitals experiencing floods in rural India [17], rural Vietnam [18], and Thailand [19]. The Ministry of Health in Sri Lanka has made several efforts to improve disaster management training; every year 15–20 doctors achieve a postgraduate diploma in health sector disaster management, and 78 doctors have completed this course so far. Additionally, in-service trainings in the last decade have included 10 on Public Health in Emergencies and Disaster Management, 11 on Sexual and Reproductive Health in emergencies, and numerous hospital-based programs. It is important to identify mechanisms whereby these types of continued education opportunities and disaster-specific trainings can reach remote areas such as Trincomalee District, so that all health professionals may feel confident and up-to-date in their knowledge of procedures or protocols. Successful strategies for flood preparedness in other settings in Asia include local meetings to promote national flood policies [20], flood trainings and plans, and human resource management including regularly updated staff contact lists and adequate staff for shift rotations [21]. Adapting existing toolkits that align with the WHO framework is another potential strategy to help healthcare facilities prepare for and mitigate the impacts of climate change [22].

The context of the three-decade civil conflict that ended in 2009 and its implications for health systems are also important. Distribution of health-related human resources has been historically uneven, with shortages in the North and East due to extended conflict there [23]. Interview respondents indicated that infrastructure is still being rebuilt in the Eastern Province, and camps for internally displaced persons (IDPs) still operate in Trincomalee District. It is important to evaluate and create future disaster-preparedness programs within the context of continued post-conflict recovery in these regions.

Overall, disaster resiliency in the Sri Lankan primary health system has both strong points and areas that need strengthening. The government provides universal healthcare, with wide geographic coverage, and Sri Lanka has some of the best health indicators in the South East Asia region [24]. Ninety-three percent of women attend 4 or more antenatal care visits (compared to an average of 54% in the 11-country WHO South-East Asia Regional Office (SEARO)), and births attended by skilled health personnel and measles immunizations are both at 99% (compared to 67% and 78%, respectively, in the WHO SEARO region) [25].

Climate change adaptation across sectors is increasingly prioritized by the government, and the link between climate change and health is acknowledged in the National Climate Change Adaptation Strategy and other national policies [26]. However, climate change is happening now and is projected to intensify. In Sri Lanka, the average temperature is expected to rise between 1.1 and 2.4°C by 2100 [27], while the population, currently with a density of 300 people per km^2^, continues to grow at an annual rate of 0.9% [28]. These trends will amplify the impacts of disasters, such as storms, floods, droughts, and sea level rise, in addition to health problems such as increased heat-related illnesses and changes in the prevalence and distribution of vector-borne diseases. These added stresses to the health system could magnify structural issues, including lack of knowledge about how to prepare for and respond to disasters, understanding of climate-sensitive health issues, and limited human resources at every level. Further, while climate change policies exist at the national level, information is not yet reaching the peripheral level of public healthcare. Current communication channels are not effective for disseminating information, and must be improved.

This research had some limitations. It only included one region of Sri Lanka. This limits the generalizability of the study, as the type and intensity of hydro-meteorological hazards vary widely throughout the country. Preparedness among health facilities may also vary widely throughout the country. Subjective responses in the study questionnaire were left to the interpretation of the respondent, and may not reflect the experiences or opinions of other facility staff. Finally, this study only includes public health facilities, and did not include private health facilities or traditional medical providers (Ayurvedic or other) in either the public or private sector.

## Conclusion

While there is a strong health system operating in Sri Lanka, there are still improvements to be made in disaster preparedness, particularly in the context of resilience to climate change. While some hazards-specific preparedness, such as flood early warning systems or disaster management training, may be necessary, it is important to also address system strains such as human resource shortages. Health system strengthening improves resilience to all hazards, but should be conducted with disasters in mind. Investments in workforce, disaster training that ensures broad access, and better resources for continuing education are all adaptation strategies that would improve disaster response and climate change mitigation now and in the future.

## Paper context

Climate change is a reality uncontested in the scientific community, and projected effects, such as more extreme weather events, will put pressure on health systems. While Sri Lanka is vulnerable to these events, no study has sought to assess the functional preparedness of public healthcare facilities for floods or other disasters. Findings suggest investments in workforce, disaster training, and better resources for continuing education are all adaptation strategies that would improve disaster response and climate change mitigation.

## Appendix. Questionnaire

**Background**

What is the name of this facility? ____________________

When was this health facility built? __________________

What type of facility is it?

☐ Medical Office of Health

☐ Teaching hospital

☐ Base hospital

☐ District hospital

☐ Peripheral hospital

☐ Other (please specify) __________________

What is the bed capacity? ___________________

Does this facility have a generator?________________

What is your current position? ____________________

How many years have you worked in your current position at this facility? ___________________

How many years have you practiced as a physician? ___________________

What is your contact information (phone number/email)? ____________________

**Flood history**

1. Has this facility experienced flooding in catchment area in last 5 years?

☐ Yes

☐ No

2. Has this facility experienced flooding on premises in last 5 years?

☐ Yes

☐ No *(please proceed to question 8)*

3. If so, how many times has your facility experienced flooding in last 5 years? ___________

4. What was the date (month and year) of last flood? ______________

5. How many days did it last? ________________

6. Duration of hospital clean-up/renovation (days)? ______________

7. During the last flood, did your facility experience:

☐ Phone lines down

☐ Internet down

☐ Power outages

☐ Road access to health facility impaired

**Flood protocols and plans**

8. Does the health facility have an overall emergency preparedness plan?

☐ Yes

☐ No *(please proceed to question 13)*

9. Is the health facility emergency plan documented in writing?

☐ Yes

☐ No

10. When was the plan last updated?

☐ Within past year

☐ Within last 1–3 years

☐ More than 3 years ago

☐ Don’t know

11. Has your facility ever conducted an exercise or drill of this emergency preparedness plan?

☐ Yes

☐ No *(please proceed to question 13)*

12. How often have you conducted an exercise or drill in the last year? _______________

13. Does your hospital have an existing flood protocol?

☐ Yes

☐ No *(please proceed to question 16)*

14. Has your facility ever conducted an exercise or drill of flood protocol?

☐ Yes

☐ No *(please proceed to question 16)*

15. If yes, how often have you conducted a drill in the last year? _______________

16. Have you or anyone in your health facility participated in a **disaster preparedness training** in the last year?

☐ Yes

☐ No *(please proceed to question 20)*

17. How many trainings have you or someone in your health facility participated in during the last five years? _________________

18. On a scale of 1 to 5, how sufficient do you think the *frequency* of training is?

**Table:**

Completely insufficient	Somewhat insufficient	Neither sufficient nor insufficient	Somewhat sufficient	Completely sufficient
				

19. On a scale of 1 to 5, how sufficient do you think the *quality* of training is?

**Table:**

Completely insufficient	Somewhat insufficient	Neither sufficient nor insufficient	Somewhat sufficient	Completely sufficient
				

**Leadership/government**

20. How often have you communicated with the *PDHS* about disaster preparedness and response within the last year?

☐ At least one time per month

☐ 6–11 times

☐ 1–5 times

☐ Have not communicated on this topic *(please proceed to question 23)*

21. How satisfied do you feel with the *frequency* of communication?

**Table:**

Completely dissatisfied	Somewhat dissatisfied	Neither satisfied nor dissatisfied	Somewhat satisfied	Completely satisfied
				

22. How satisfied do you feel with the *quality* of communication?

**Table:**

Completely dissatisfied	Somewhat dissatisfied	Neither satisfied nor dissatisfied	Somewhat satisfied	Completely satisfied
				

23. How often have you communicated with the *RDHS* about disaster preparedness and response within the last year?

☐ At least one time per month

☐ 6–11 times

☐ 1–5 times

☐ Have not communicated on this topic *(please proceed to question 26)*

24. How satisfied do you feel with the *frequency* of communication?

**Table:**

Completely dissatisfied	Somewhat dissatisfied	Neither satisfied nor dissatisfied	Somewhat satisfied	Completely satisfied
				

25. How satisfied do you feel with the *quality* of communication?

**Table:**

Completely dissatisfied	Somewhat dissatisfied	Neither satisfied nor dissatisfied	Somewhat satisfied	Completely satisfied
				

26. Are instructive documents on disaster preparedness made available to your facility from *PDHS*?

☐ Yes

☐ No *(please proceed to question 30)*

27. If yes, how satisfied are you with the *frequency* with which you receive these documents?

**Table:**

Completely dissatisfied	Somewhat dissatisfied	Neither satisfied nor dissatisfied	Somewhat satisfied	Completely satisfied
				

28. How satisfied are you with the *quality* of these documents?

**Table:**

Completely dissatisfied	Somewhat dissatisfied	Neither satisfied nor dissatisfied	Somewhat satisfied	Completely satisfied
				

29. Are instructive documents on disaster preparedness made available to your facility from *RDHS*?

☐ Yes

☐ No *(please proceed to question 32)*

30. If yes, how satisfied are you with the *frequency* with which you receive these documents?

**Table:**

Completely dissatisfied	Somewhat dissatisfied	Neither satisfied nor dissatisfied	Somewhat satisfied	Completely satisfied
				

31. How satisfied are you with the *timeliness* with which you receive these documents?

**Table:**

Completely dissatisfied	Somewhat dissatisfied	Neither satisfied nor dissatisfied	Somewhat satisfied	Completely satisfied
				

32. Are instructive documents on disaster preparedness made available to your facility from the *Disaster Management Centre*?

☐ Yes

☐ No *(please proceed to question 34)*

33. If yes, how satisfied are you with the *frequency* with which you receive these documents?

**Table:**

Completely dissatisfied	Somewhat dissatisfied	Neither satisfied nor dissatisfied	Somewhat satisfied	Completely satisfied
				

34. How satisfied are you with the *timeliness* with which you receive these documents?

**Table:**

Completely dissatisfied	Somewhat dissatisfied	Neither satisfied nor dissatisfied	Somewhat satisfied	Completely satisfied
				

**Healthcare financing**

35. How satisfied are you with the level of funding this health facility receives for flood and storm preparedness and response?

**Table:**

Completely dissatisfied	Somewhat dissatisfied	Neither satisfied nor dissatisfied	Somewhat satisfied	Completely satisfied
				

36. Do you have funds for weather emergencies of any sort in your annual operating budget?

☐ Yes

☐ No *(please proceed to question 38)*

37. If yes, are any of these emergency funds specifically for flood preparedness or response?

☐ Yes

☐ No

**Information management**

38. Do you report flood events?

☐ Yes

☐ No

39. If so, to whom? *(Please check all that apply)*

☐ RDHS

☐ PDHS

☐ Disaster Management Centre

☐ Disaster Preparedness and Response Unit

☐ Other Ministry of Health entity

☐ Other entity (please specify) ____________________

**Health workforce**

40. On average, how many patients does your facility see per day? __________

41. Do you feel that you have enough staff to successfully perform normal service delivery during a weather emergency?

☐ Yes

☐ No

42. If your facility has experienced past floods, were staff absent due to the flood?

☐ Yes

☐ No

☐ Not applicable (no prior floods)

43. If so, did this create problems in service delivery?

☐ Yes

☐ No

☐ Not applicable (no prior floods)

44. How many clinical staff (doctors, nurses, midwives) work at this facility? _______________

45. How many total staff work at this facility? ____________

**Medical products, technologies**

46. Are there first-aid kits available at your health facility?

☐ Yes

☐ No

47. Does the health facility have a system in place for emergency procurement of supplies?

☐ Yes

☐ No

48. How long does the procurement of supplies take under emergency conditions?

☐ No delay in procurement

☐ 1–6 days longer than usual

☐ 1–2 weeks longer than usual

☐ 2 weeks or more longer than usual

☐ Other (please specify) _______________

49. Do you think that your health facility has enough medical equipment and supplies for normal service operation during a flood or storm?

☐ Yes

☐ No

**Service delivery**

50. Are there ever times when preventive or curative services are not available during storms or floods?

☐ Yes

☐ No *(please proceed to question 52)*

51. If so, what are the reasons? (please check all that apply)

☐ Staff absent from facility

☐ Lack of supplies

☐ Damage to facility medical equipment

☐ Damage to building infrastructure

☐ Impaired or blocked road access to health facility

☐ Power outages

☐ Other (please specify) _______________________

**Institutional safety score**

52. How prepared do you feel your health facility is for an *extreme flood event*?

**Table:**

Completely unprepared	Somewhat unprepared	Neither prepared nor unprepared	Somewhat prepared	Completely prepared
				

53. How prepared do you feel your health facility is for *any type of disaster*?

**Table:**

Completely unprepared	Somewhat unprepared	Neither prepared nor unprepared	Somewhat prepared	Completely prepared
				

**Climate change**s

54. Do you think flood and storm events happen more frequently than they used to?

☐ Yes

☐ No

☐ Don’t know

55. Do you think flood and storm events are stronger or more intense than they used to be?

☐ Yes

☐ No

☐ Don’t know

56. Do you think that climate change is occurring in Sri Lanka?

☐ Yes

☐ No

☐ Don’t know

57. Do you think changes in climate can affect human health?

☐ Yes

☐ No

☐ Don’t know

58. Have you received any disaster management communication or documents from the PDHS or RDHS that includes information or guidance on climate change?

☐ Yes

☐ No

☐ Don’t know
